# Linguistic validation and reliability of the Brazilian Portuguese version of the Composite Autonomic Symptom Score 31 (COMPASS 31)

**DOI:** 10.1007/s10072-026-09039-8

**Published:** 2026-04-15

**Authors:** Maíra D. Correia, Fabiana C. da Silva, Robert Schleip, Wolfgang Singer, Ronaldo H. Cruvinel-Júnior, Marcelo Faria Silva

**Affiliations:** 1 Physical Therapy Department, Federal University of Health Sciences of Porto Alegre, Porto Alegre, Brazil; 2 Department of Physical Therapy, Speech, and Occupational Therapy, School of Medicine, University of São Paulo, São Paulo, Brazil; 3 Conservative and Rehabilitative Orthopedics, TUM School of Medicine and Health, Technical University of Munich, Munich, Germany; 4 Department for Medical Professions, Diploma Hochschule, Bad Sooden-Allendorf, Germany; 5 Experimental Anesthesiology, Ulm University, Ulm, Germany; 6 Department of Neurology, Mayo Clinic, Rochester, MN USA

**Keywords:** Autonomic dysfunction, Dysautonomia, COMPASS-31, Validation study

## Abstract

**Background:**

The Composite Autonomic Symptom Score 31 (COMPASS-31) is a validated self-report questionnaire designed to assess autonomic symptoms across six domains. A Brazilian Portuguese version has not yet been formally validated.

**Methods:**

Following a standardized Mayo Clinic linguistic validation protocol, the COMPASS-31 was translated into Brazilian Portuguese. Twenty-one patients with disorders associated with autonomic symptoms and nine healthy controls completed both the English and Portuguese versions in randomized order, with a six-week interval between administrations. Reliability was assessed using kappa coefficients and Bland–Altman analysis. Validity was evaluated by comparing scores between patients and controls and by correlating the Portuguese and English versions.

**Results:**

The Brazilian Portuguese version of the COMPASS-31 demonstrated good test–retest reliability and a strong correlation with the original English version (*r* = 0.784, *p* < 0.0001). Patients exhibited significantly higher total scores than healthy controls. Bland–Altman analysis showed good agreement, with 93.4% of values falling within the limits of agreement.

**Conclusions:**

The Brazilian Portuguese version of the COMPASS-31 is a valid and reliable instrument for assessing autonomic symptoms in both clinical and research settings.

**Supplementary Information:**

The online version contains supplementary material available at 10.1007/s10072-026-09039-8.

## Introduction

The autonomic nervous system (ANS) is a complex network responsible for the involuntary regulation of essential visceral functions. It comprises the sympathetic, parasympathetic, and enteric systems, which operate in an integrated manner across the central and peripheral nervous systems [1]. When disrupted, the ANS can give rise to a broad spectrum of clinical manifestations involving cardiovascular, urogenital, gastrointestinal, thermoregulatory, sudomotor, and pupillomotor functions [2].

Autonomic symptoms are frequently observed in individuals with neurological disorders such as movement disorders, neuro-immunological conditions, cerebrovascular diseases, neurodegenerative disorders, and spinal cord injuries [3–9]. Despite their prevalence, these symptoms often go unrecognized in routine clinical practice [10]. Diagnostic challenges arise from their nonspecific nature, frequent overlap with other conditions, and the absence of standardized, accessible tools. As a result, early detection is limited, effective treatment is delayed, and patient quality of life is adversely affected [11, 12].

To address these challenges, structured assessment tools have been developed. Among them, the Composite Autonomic Symptom Score 31 (COMPASS-31) stands out as a validated, self-administered questionnaire specifically designed to capture the breadth of autonomic symptoms [13]. Derived from the more extensive 169-item Autonomic Symptom Profile (ASP) [2] and the 84-item COMPASS questionnaire [13], the COMPASS-31 condenses this evaluation into 31 items across six domains: orthostatic intolerance, vasomotor, secretomotor, gastrointestinal, bladder, and pupillomotor symptoms. Its shorter format not only makes it more practical for patients but also simplifies scoring for clinicians, without compromising accuracy or comprehensiveness [13].

Since its introduction, the COMPASS-31 has been widely adopted in both clinical and research contexts, proving sensitive, reliable, and adaptable across cultures. Translation and validation studies have been successfully conducted in several countries, including Denmark [14], Turkey [15], Italy [16], and Germany [10]. These cross-cultural validations underscore the instrument’s robustness and international applicability.

Nevertheless, a validated Brazilian Portuguese version of the COMPASS-31 remains unavailable. This gap hinders the standardization of clinical and epidemiological data, limits the early recognition of autonomic dysfunction, and restricts its integration into clinical and research practices in Brazil.

*This study is part of an international effort coordinated by the Autonomic Research Group at the Mayo Clinic*,* aimed at producing expert translations of the COMPASS-31 into multiple languages and evaluating their linguistic and psychometric validity. Previous validation studies*,* including the Danish* [14], *German* [10], *Turkish* [15] *and Italian* [16] *versions*,* have followed a standardized protocol established by this group*,* ensuring semantic equivalence*,* reliability*,* and cross-cultural applicability. Our Brazilian Portuguese validation adheres to this same methodology*,* contributing to the broader global initiative to facilitate consistent autonomic symptom assessment across diverse populations.*

Therefore, the present study aims to perform the linguistic validation and reliability testing of the COMPASS-31 in Brazilian Portuguese, following the standardized protocol established by the Mayo Clinic. Providing an accessible, non-invasive, and low-cost tool, the validated version is expected to enhance clinical decision-making, advance research, and support integrative patient care in the Brazilian context.

## Methods

### Translation of the COMPASS-31 from the US English into Brazilian Portuguese

The linguistic validation of the COMPASS-31 into Brazilian Portuguese was a collaborative effort between the Autonomic Research Group from Mayo Clinic and the authors of this study. The Mayo Clinic, in partnership with the translation company Mapi (Lyon, France), was responsible for coordinating and providing the translation of the instrument. The process was performed with a rigorous methodology to ensure the translated questionnaire was conceptually equivalent to the original U.S. English version, culturally relevant, and easily understood by the Brazilian population (Supplementary Material 1).

This process for the Brazilian Portuguese version involved several steps. Initially, the questionnaire was translated from the original into Brazilian Portuguese by two qualified translators. Subsequently, a single back-translation was performed by one qualified translator. A clinical specialist then reviewed the translated version, followed by cognitive interviews with five healthy individuals to ensure its clarity and cultural relevance. Mapi centrally coordinated the entire process and performed a quality control check at each step, under the supervision of a consultant in Brazil. This certified linguistic validation confirms that the final Brazilian Portuguese version is suitable for use in clinical studies.

### Study participants

The study was developed in the University of Health Sciences of Porto Alegre, Rio Grande do Sul, Brazil. This cohort included 21 patients with disorders that could be associated with autonomic symptoms and 9 age- and gender-matched healthy controls. To avoid focusing on patients with autonomic dysfunction in only one or two of the six COMPASS-31 domains, we enrolled individuals with a variety of different diseases. *The sample was selected by convenience through contact with physicians who treat individuals diagnosed with dysautonomia. In addition*,* communication channels such as social media were used to publicize the study and support participant recruitment.*

All participants were bilingual, being native speakers of Brazilian Portuguese and fluent in English. *Both language versions were administered to the same participants*,* a methodology commonly used in linguistic validation studies to assess cross-language equivalence. This approach has been previously adopted in COMPASS-31 validation studies in other languages* [10, 14, 16] *and represents a standard step prior to broader validation in monolingual populations.*

Ethical approval for the study was granted by the Federal University of Health Sciences of Porto Alegre ethics committee on October,10, 2024 (Approval No:7.148.721), and all participants provided informed consent in accordance with the Declaration of Helsinki. All data was analyzed confidentially and in compliance with the Brazilian General Data Protection Law (LGPD), Law No. 13,709, of August 14, 2018 [17].

The 30 study participants completed the COMPASS-31 questionnaire twice, within an interval of approximately six weeks (± one week). They received both the Brazilian Portuguese and US English versions. *This sample size is consistent with previous linguistic validation studies of the COMPASS-31*,* which have included similar numbers of participants* [10, 14, 16]. The order of completing the questionnaires was determined by a randomized entry number provided by the Mayo Clinic in Rochester, MN, USA. To ensure consistency, we only enrolled patients with a stable clinical disease course and no major changes in their management planned for the 6-week period.

All patients and controls underwent a series of standardized autonomic cardiovascular tests to confirm or exclude autonomic dysfunction. These tests, which have been previously described in the literature, included the Active Stand Test for orthostatic hypotension [18] and Heart Rate Variability [19]. Heart rate data was collected using a Polar V800 heart rate monitor [19] and processed with Kubios HRV software [20].

Participants with diagnosed autonomic dysfunction of varying degrees were eligible for the study, provided they were between 18 and 79 years old and could read and comprehend the English language. All participants needed to be clinically stable, with no changes in their medication regimen for at least one month before the initial questionnaire and one month after, until the second application. They also had to be able to move, lie down, and stand up on their own to perform the Active Standing Test. Exclusion criteria included severe dysautonomia that prevented questionnaire completion, a lack of English reading comprehension, an unstable treatment regimen, or progressive disease. Additionally, minors and individuals with compromised functional or cognitive independence were excluded.

*While our participant cohort includes heterogeneous conditions*,* each has documented or suspected autonomic involvement that justifies assessment with a broad autonomic symptom tool such as COMPASS-31. This approach is consistent with previous validation studies*,* which have also included clinically diverse populations rather than restricting samples to classical autonomic disorders* [14, 15].

### Data collection and scoring algorithm of COMPASS-31

The COMPASS-31 questionnaire is an instrument with 31 items that evaluate six distinct domains of autonomic symptoms. These domains are orthostatic intolerance (4 items), vasomotor (3 items), secretomotor (4 items), gastrointestinal (12 items), bladder (3 items), and pupillomotor (5 items). Detailed information on the items is available in the original publication of the U.S. English Version of COMPASS-31 [13].

For scoring, each answer is first assigned a raw score, and the sum of all raw scores within a domain constitutes the domain’s sub-raw score. This sub-raw score is then converted into a weighted sub-score by multiplying it by a weighting factor, which is derived from the relevance of each domain for assessing autonomic function [13]. The sum of the six weighted sub-scores provides a total weighted score that ranges from 0 to 100. A score of 0 indicates an absence of autonomic symptoms, while a score of 100 reflects the most severe symptoms [13]. All collected data was uploaded to Redcap (Vanderbilt University, Nashville, Tennessee), a secure online database system at the Mayo Clinic in Rochester, MN, USA.

### Statistical analysis

Statistical analyses were conducted using SPSS v.26 (IBM Corp., Armonk, NY, USA). Descriptive analyses were performed to compare clinical characteristics between patients and healthy controls. Categorical data were presented as frequencies (n) and percentages (%), while numerical data were presented as mean (standard deviation). The normality of the distribution of variables was assessed using Shapiro-Wilk tests. Continuous data from independent groups were analyzed using the Unpaired Student’s t-test. Categorical data were compared using the Chi-square test.

The kappa coefficient was used to assess the reliability of the 31 items. Kappa coefficients were interpreted as follows: 0.81-1.00 very good agreement; 0.61–0.80 good agreement; 0.41–0.60 moderate agreement; 0.21–0.40 fair agreement; 0.00-0.20 poor agreement.

The validity of the COMPASS-31 was evaluated through Bland-Altman analysis to assess test-retest reliability within a 95% confidence interval. Furthermore, validity was examined by comparing the total and domain-specific scores between patients and healthy controls across both versions of the questionnaire using the Mann-Whitney U test. Descriptive statistics for the scores and sub-scores were also calculated. A p-value of < 0.05 was considered statistically significant.

To assess the reliability of the translated, Brazilian Portuguese version of the COMPASS-31, we correlated the total weighted scores and the weighted sub-scores of the Brazilian Portuguese COMPASS-31 version with the respective weighted scores in the original English version, using Pearson’s correlation for normally distributed scores and Spearman’s rank correlation for non-normally distributed scores.

## Results

A total of 30 individuals were included in the study. The patient group was composed of 21 individuals (16 women, 41.10 ± 16.50 years), while the healthy control group consisted of 9 individuals (5 women, 41.22 ± 9.96 years). As detailed in Table 1, no significant differences in age or gender were observed between the two groups. All participants successfully completed both scales within the allotted time. The diagnoses for the 21 patients were diverse and included Irritable Bowel Syndrome, Hashimoto’s thyroiditis, Vasovagal syndrome, Ankylosing Spondylitis, Rheumatoid Arthritis, Chronic low back pain, Diabetic neuropathy, Hypothyroidism, Sleep disorder, Generalized Anxiety Disorder, Multiple Sclerosis, and Parkinson’s Disease (Table 1).

**Table 1:** Demographic and clinical characteristics of the study sample

Age (years)	Total (*n* = 30)	Patients (*n* = 21)	Healthy Controls (*n* = 9)	*p* value
	41.13 (14.67)	41.10 (16.50)	41.22 (9.96)	0.983^a^
Sex, n (%)				0.258^b^
Men	9 (30%)	5 (23.8%)	4 (44.4%)	
Women	21 (70%)	16 (76.2%)	5 (55.6%)	
Diagnosis, n (%)				
Irritable Bowel Syndrome		3 (14.2%)		
Hashimoto’s thyroiditis		1 (4.8%)		
Vasovagal syndrome		2 (9.5%)		
Ankylosing Spondylitis		1 (4.8%)		
Rheumatoid Arthritis		1 (4.8%)		
Chronic low back pain		3 (14.2%)		
Diabetic neuropathy		1 (4.8%)		
Hypothyroidism		1 (4.8%)		
Sleep disorder		4 (19.0%)		
Generalized Anxiety Disorder		2 (9.5%)		
Multiple Sclerosis		1 (4.8%)		
Parkinson Disease		1 (4.8%)		

Age and gender distribution did not differ between patients and healthy controls. Data are expressed as mean (standard deviation) or as number (percentage). a = Unpaired Student’s t-test; b = Chi-squared test

The kappa coefficient was used to analyze the reliability of the 31 items. The kappa analysis indicated that 4 items (13%) demonstrated good agreement, 17 items (54.8%) showed moderate agreement, 5 items (16.1%) showed fair agreement, and 5 items (16.1%) showed poor agreement (Table 2). Items with lower kappa values may reflect symptom fluctuation rather than linguistic inconsistency, particularly in domains with episodic manifestations.

**Table 2:** Kappa Analysis based on responses of all 30 study participants

Question	Kappa	Question	Kappa	Question	Kappa
Question 1	0.769	Question 12	0.450	Question 23	0.429
Question 2	0.539	Question 13	0.371	Question 24	0.455
Question 3	0.556	Question 14	0.359	Question 25	0.194
Question 4	0.412	Question 15	0.784	Question 26	0.498
Question 5	0.524	Question 16	0.638	Question 27	0.354
Question 6	0.524	Question 17	0.403	Question 28	0.497
Question 7	0.273	Question 18	0.479	Question 29	0.081
Question 8	0.474	Question 19	0.545	Question 30	0.088
Question 9	0.561	Question 20	0.714	Question 31	0.177
Question 10	0.412	Question 21	0.268		
Question 11	0.080	Question 22	0.574		

0.81-1.00 very good agreement; 0.61–0.80 good agreement; 0.41–0.60 moderate agreement; 0.21–0.40 fair agreement; 0.00-0.20 poor agreement

### Comparison of COMPASS-31 scores between patient and healthy control groups

When comparing the subscale and total scores of the COMPASS-31 scale between patient and healthy control groups, statistically significant differences were observed in several domains, depending on the language version (Table 3). In the Brazilian Portuguese version, patients scored significantly higher than healthy controls in the Orthostatic Intolerance, Gastrointestinal, and Pupillomotor domains, as well as in the total COMPASS- 31 score (*p* = 0.013) (Table 3). In the English version, significant differences were found in the Secretomotor, Gastrointestinal, and Pupillomotor domains, and also in the total COMPASS-31 score (*p* = 0.034) (Table 3). No significant differences were identified in the Vasomotor or Bladder domains for either version. These findings confirm that, as expected, patients presented higher levels of autonomic symptoms compared to healthy controls (Table 3).

**Table 3:** Comparison of subscale and total scores between patient and healthy control groups

Domain	Version	Patients (*n* = 21)	Healthy Controls (*n* = 9)	*p* value
Orthostatic Intolerance Score	English	16 (12–20)	0 (0–18)	0.175
	Portuguese	16 (10–18)	0 (0–8)	0.011
Vasomotor Score	English	0 (0–0)	0 (0–0)	0.287
	Portuguese	0 (0–1.67)	0 (0–0)	0.140
Secretomotor Score	English	2.14 (0–6.43)	0 (0–2.14)	0.034
	Portuguese	0 (0–2.14)	0 (0–3.21)	0.884
Gastrointestinal Score	English	8.93 (4.91–10.27)	4.46 (0.89–7.14)	0.024
	Portuguese	7.14 (4.01–10.71)	1.79 (1.34–5.36)	0.013
Bladder Score	English	0 (0–1.66)	0 (0–1.11)	0.476
	Portuguese	0 (0–1.11)	0 (0–0.55)	0.412
Pupillomotor Score	English	2 (1.33–2.67)	1 (0–1.67)	0.024
	Portuguese	2 (1–2.67)	1 (0–1.66)	0.013
COMPASS-31 Score	English	29.50 (21.66–36.65)	7.72 (2.29–27.35)	0.018
	Portuguese	25.95 (21.07–32.45)	9.28 (2.91–17.26)	0.001

Median (25–75 Percentile). Mann-Whitney U test performed for all comparisons

### Validity analysis

**Fig. 1:**
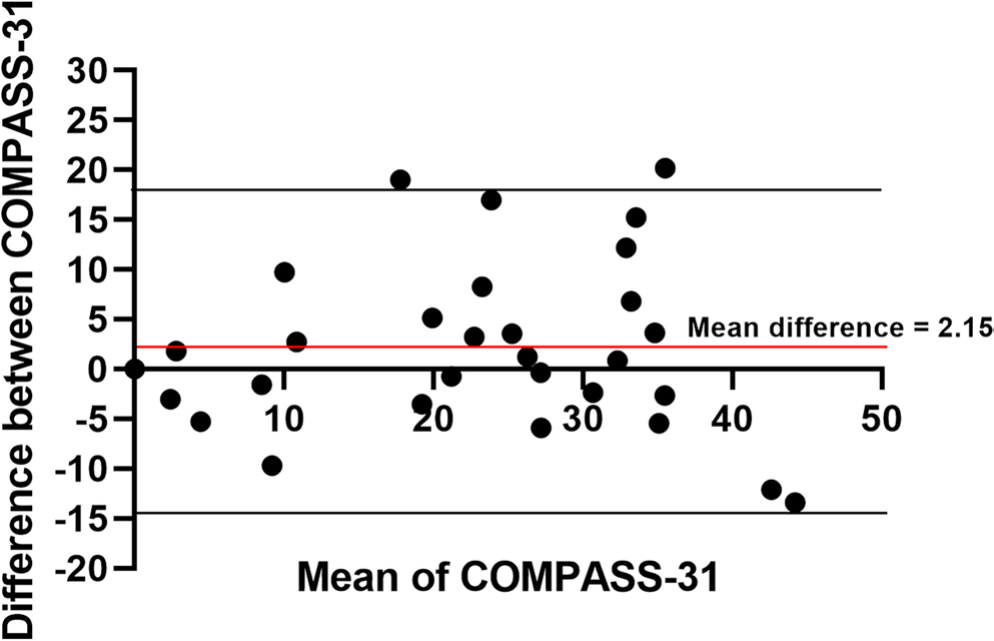
Test-retest reliability of the English and Brazilian Portuguese versions of the COMPASS-31 questionnaire evaluated by a Bland Altman plot

The Bland-Altman plot demonstrated good test-retest reliability with no consistent bias of one version of the COMPASS-31 questionnaire versus the other (Fig. 1). It was observed that only 2 patients (6.6%) were outside the 95% confidence interval (Fig. 1).

### Reliability of the Portuguese version of COMPASS-31

Among the 30 study participants, the Brazilian Portuguese version of the COMPASS-31 showed a significant and positive correlation with the original English version for the total weighted score (coefficient = 0.784, *p* < 0.0001), as well as for most weighted sub-scores: orthostatic intolerance (coefficient = 0.640, *p* = 0.0001), vasomotor (coefficient = 0.496, *p* = 0.005), gastrointestinal (coefficient = 0.864, *p* < 0.0001), bladder (coefficient = 0.661, *p* < 0.0001), and pupillomotor domains (coefficient = 0.603, *p* = 0.0004). The only exception was the secretomotor domain, which did not reach statistical significance (coefficient = 0.320, *p* = 0.084) (Table 4).

**Table 4:** Correlation between total and sub-domain scores in the Portuguese and English COMPASS-31 versions assessed in 30 study participants

Domain	Correlation between Brazilian Portuguese and English COMPASS-31 scores	
	Correlation coefficient	*p*-value
Orthostatic Intolerance Score	0.640^b^	0.0001
Vasomotor Score	0.496^b^	0.005
Secretomotor Score	0.320^b^	0.084
Gastrointestinal Score	0.864^a^	< 0.0001
Bladder Score	0.661^b^	< 0.0001
Pupillomotor Score	0.603^a^	0.0004
COMPASS-31 Score	0.784^a^	< 0.0001

a = Pearson’s correlation, b = Spearman’s rank correlation. COMPASS-31 Composite Autonomic Symptom Score 31

## Discussion

This study successfully validated the Brazilian Portuguese version of the COMPASS-31 questionnaire, demonstrating that it is a reliable and valid instrument for assessing autonomic symptoms in the Brazilian population. The validation process followed a rigorous methodology, including a standardized forward and back-translation procedure in collaboration with the Mayo Clinic, ensuring the conceptual and cultural equivalence of the translated version to the original U.S. English version.

Our findings indicate a high test-retest reliability of the Brazilian Portuguese COMPASS-31, which is consistent with the results from other validation studies of the COMPASS-31 in different languages, such as Italian [16], Danish [14], German [10] and Turkish [15]. We observed a strong correlation between the total scores of the Brazilian Portuguese and the original English versions of the questionnaire (*r* = 0.784; *p* < 0.0001), similar to the high correlations reported in the German [10] and Italian [16] validation studies. This strong correlation confirms the linguistic and cultural validity of the translated instrument. The lack of significant correlation in the secretomotor domain may be related to the low variance observed in this sample and the small number of controls.

Furthermore, the Bland-Altman analysis demonstrated good agreement between the two versions, with only a small proportion of values (6.6%) falling outside the confidence interval, which supports the reliability of our translation. Interestingly, these outliers corresponded to just two participants (one patient and one healthy control), and their deviation can likely be attributed to transient changes in autonomic symptoms reported between assessments. Similar findings were observed in the Turkish validation study [15], where only four patients (4.4%) fell outside the 95% confidence interval, and the discrepancies were likewise explained by temporary fluctuations in autonomic symptoms between test and retest evaluations. *Despite the overall strong reliability results*,* some individual items demonstrated fair or poor kappa values. This finding is likely related to the low prevalence of certain autonomic symptoms in our sample*,* as kappa statistics are known to be sensitive to imbalanced response distributions. Additionally*,* the subjective nature of symptom reporting may contribute to variability at the item level. Importantly*,* these limitations were restricted to specific items and did not affect the overall reliability of the questionnaire*,* as demonstrated by the high internal consistency and robust test–retest reliability of domain and total scores.*

The Brazilian Portuguese COMPASS-31 also demonstrated good construct validity by effectively discriminating between patients with possible autonomic symptoms and healthy controls. The patient group had significantly higher total scores compared to the control group, a finding that is in line with the Danish [14], German [10], and Italian [16] studies, which also reported significantly higher scores in patient populations. This confirms the questionnaire’s ability to detect and quantify autonomic symptoms in a clinical context.

This study is integrated into a global initiative led by the Research Autonomic Group at the Mayo Clinic, whose purpose is to produce expert translations of the COMPASS-31 into multiple languages and to evaluate the validity of these versions. Along with our institution, ten other centers across the world (including China, Japan, South Korea, Germany, France, Spain, the Netherlands, Denmark, Sweden, and Italy) are collaborating. The collective findings are expected to provide a stronger foundation for determining the clinical usefulness of this questionnaire.

One of the main strengths of our study is the rigorous, multi-step translation and cultural adaptation process, which included cognitive interviews with a target population sample. This ensures that the items are not only accurately translated but are also easily understood and relevant within the Brazilian cultural context. However, we must also acknowledge one limitation of our study. Although the sample size is modest, it is comparable to initial validation studies in other languages and adequate for preliminary linguistic validation and reliability testing. *Additionally*,* although standardized autonomic function tests were performed for clinical characterization*,* no formal correlation analyses with COMPASS-31 scores were conducted. Future studies should explore the association between COMPASS-31 scores and objective autonomic function tests to further establish criterion validity.*

Future research involving a larger cohort of patients would be beneficial to establish normative data for the Brazilian population.

The availability of a validated version of the COMPASS-31 in Brazilian Portuguese is a significant step forward for clinical practice and research in Brazil. It provides a non-invasive, low-cost, and easy-to-use tool for the early detection and monitoring of autonomic dysfunction in various neurological and non-neurological conditions. This will facilitate the standardization of clinical data, improve patient care, and foster national and international research collaborations.

## Conclusion

In conclusion, the Brazilian Portuguese COMPASS-31 is a valid and reliable instrument. The development of this tool closes a significant gap for clinical practice and research in Brazil, providing a standardized, non-invasive, and accessible method for quantifying autonomic symptom burden. Its availability will facilitate the early identification of autonomic dysfunction, help monitor disease progression and treatment response, and finally enable its use as a validated outcome measure in clinical trials conducted within Brazil and internationally.

## Supplementary Information

Below is the link to the electronic supplementary material.

Supplementary Material 1

## Data Availability

Anonymized patient data will be made available to qualified investigators upon reasonable request.
