# Supplementary material for: Linguistic validation and reliability of the Brazilian Portuguese version of the Composite Autonomic Symptom Score 31 (COMPASS 31)
Source: Neurol Sci. 2026 Apr 15;47(5):421. doi: 10.1007/s10072-026-09039-8 (PMC13079522; doi:10.1007/s10072-026-09039-8)
Supplement: Supplementary file 1 — Supplementary Material 1 [file 10072_2026_9039_MOESM1_ESM.pdf]

Mapi
Insight for solutions

LINGUISTIC VALIDATION CERTIFICATE
PAPER VERSION OF COMPOSITE AUTONOMIC SYMPTOM SCALE 31 (COMPASS31)

This is to certify that Mapi conducted the linguistic validation of the paper version of the **COMPASS31** into the languages listed on the following page.

The aim of a linguistic validation is to obtain translations that are:
- conceptually equivalent to the original and comparable across languages;
- culturally relevant to the context of the target country;
- easily understood by the people to whom the translated instrument is administered.

This is achieved using a rigorous methodology[1] involving:
- a process which comprises several steps (see diagram on the last page of this document);
- the collaboration of the instrument's developer and a skilled team recruited by Mapi in the target country which is headed by a consultant with knowledge of and experience in the field of Patient-Reported Outcomes;
- a centralized review process coordinated by Mapi.

The specific methodology used to produce these versions of the **COMPASS31** is summarized in the table on the following page. For each language, the linguistic validation process was coordinated and supervised by a consultant in the target country under the guidance of Mapi who performed a quality control and discussed the translation decisions with the consultant at each step of the process.

Ana Bayles
Managing Director
Mapi - Linguistic Validation

Mapi SAS
SAS au capital de 434 700 euros
27 rue de la Villette
69003 LYON
Tél. 04 72 13 66 67 - Fax 04 72 13 69 50
RCS Lyon 378 472 872 - TVA n° FR 66 378 472 872

Date: 21 July 2014

[1] **References:**
- Acquadro C., Jambon B., Ellis D. and Marquis P. Language and translation issues. In Spilker B, ed. Quality of Life and Pharmacoeconomics in Clinical Trials. Philadelphia: Lippincott-Raven Publishers, 1996: 575-585.
- Linguistic Validation Manual for Health Outcomes Assessments. Acquadro C, Conway K, Giroudet C, Mear I. Second Edition - MAPI Institute, Lyon, France, January 2012 - ISBN: 2-9522021-0-9

© Copyright 2006 - 2014. Mapi. All Rights Reserved.

z:\operations\questionnaires\question a-f\compass (composite autonomic symptom scale)\09_translation_certificates\7502-project-specific\02_7502-project-specific_compass31_trans-certif_21-07-2014_ggr.doc

LINGUISTIC VALIDATION STEPS<br>COUNTRIES	LANGUAGES	**Forward Translation Step**<br>(2 translations by qualified translators ➔ reconciliation)	**Backward Translation Step**<br>(1 translation by a qualified translator)	**Adaptation Step**<br>(Review and adaptation of the mother language version to context of the target country)	**Clinician's Review Step**<br>(Sponsor-appointed clinician)	**Cognitive Interview Step**<br>(on 5 healthy individuals)
**Brazil**	**Portuguese**	☑	☑	N/A	☐	☑
**France**	**French**	☑	☑	N/A	☐	☑
**Germany**	**German**	☑	☑	N/A	☐	☑
**Italy**	**Italian**	☑	☑	N/A	☐	☑
**Japan**	**Japanese**	☑	☑		☑	☑
**Netherlands**	**Dutch**	☑	☑	N/A	☐	☑
**South Korea**	**Korean**	☑	☑	N/A	☐	☑
**Spain**	**Spanish**	☑	☑	N/A	☐	☑
**Sweden**	**Swedish**	☑	☑	N/A	☐	☑
**United States**	**English**	**Original instrument**				

N/A: Not applicable ☑: Step performed ☐: Step not performed

© Copyright 2006 - 2013. Mapi. All Rights Reserved.

The linguistic validation process

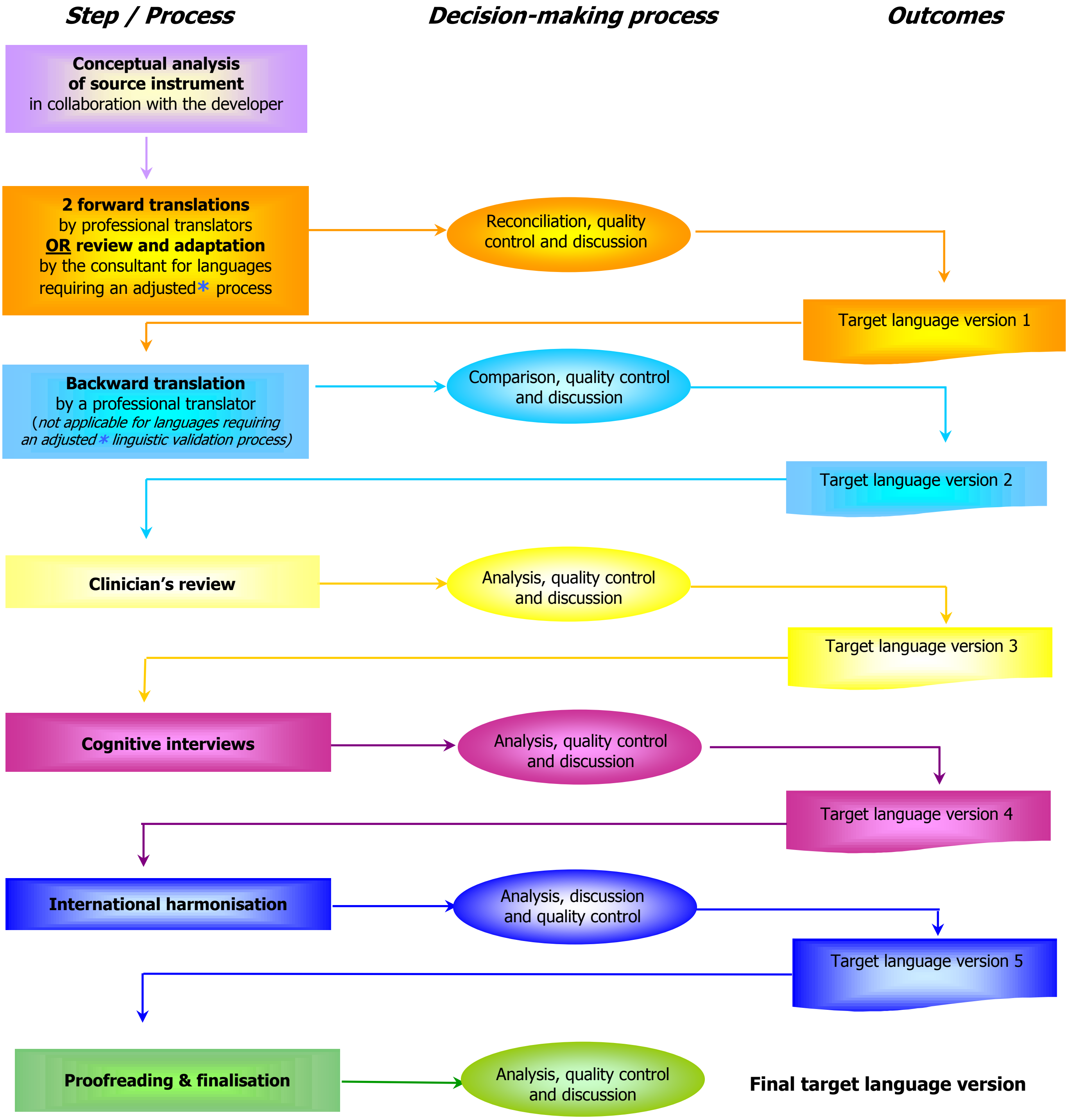

* For some languages that are close to one another (e.g. British and American English; French for France and Belgium), the complete standard linguistic validation process outlined above with *forward* and *backward* translation steps may not be appropriate. For such cases, an **adjusted** linguistic validation process has been established. The *forward* and *backward* translation steps are replaced by a review and adaptation step, where the work is based on a version considered as the "mother language" version. The subsequent steps are identical to those used in the standard linguistic validation process.

© Copyright 2006 - 2013. Mapi. All Rights Reserved.
